# Dixon MRI-based quantitative parameters of extraocular muscles, intraorbital fat, and lacrimal glands for staging thyroid-associated ophthalmopathy

**DOI:** 10.1186/s13244-024-01693-w

**Published:** 2024-06-09

**Authors:** Xiong-Ying Pu, Lu Chen, Hao Hu, Qian Wu, Wen-Hao Jiang, Jin-Ling Lu, Huan-Huan Chen, Xiao-Quan Xu, Fei-Yun Wu

**Affiliations:** 1 Department of Radiology, The First Affiliated Hospital of Nanjing Medical University, Nanjing, China; 2 Department of Endocrinology, The First Affiliated Hospital of Nanjing Medical University, Nanjing, China

**Keywords:** Thyroid-associated ophthalmopathy, Extraocular muscle, Lacrimal gland, Intraorbital fat, Dixon magnetic resonance imaging

## Abstract

**Objective:**

To investigate the value of Dixon magnetic resonance imaging (MRI)-based quantitative parameters of extraocular muscles (EOMs), intraorbital fat (IF), and lacrimal glands (LGs) in staging patients with thyroid-associated ophthalmopathy (TAO).

**Methods:**

Two hundred patients with TAO (211 active and 189 inactive eyes) who underwent Dixon MRI for pretreatment evaluation were retrospectively enrolled and divided into training (169 active and 151 inactive eyes) and validation (42 active and 38 inactive eyes) cohorts. The maximum, mean, and minimum values of the signal intensity ratio (SIR), fat fraction (FF), and water fraction (WF) of EOMs, IF, and LGs were measured and compared between the active and inactive groups in the training cohort. Binary logistic regression analysis, receiver operating characteristic curve analysis, and the Delong test were used for further statistical analyses, as appropriate.

**Results:**

Compared with inactive TAOs, active TAOs demonstrated significantly greater EOM-SIR_max_, EOM-SIR_mean_, EOM-SIR_min_, IF-SIR_max_, IF-SIR_mean_, LG-SIR_max_, LG-SIR_mean_, EOM-WF_mean_, EOM-WF_min_, IF-WF_max_, IF-WF_mean_, and LG-WF_mean_ and lower EOM-FF_max_, EOM-FF_mean_, IF-FF_mean_, IF-FF_min_, and LG-FF_mean_ values (all *p* < 0.05). The EOM-SIR_mean_, LG-SIR_mean_, and LG-FF_mean_ values were independently associated with active TAO (all *p* < 0.05). The combination of the EOM-SIR_mean_, LG-SIR_mean_, and LG-FF_mean_ values showed better performance than the EOM-SIR_mean_ value alone in staging TAO in both the training (AUC, 0.820 vs 0.793; *p* = 0.016) and validation (AUC, 0.751 vs 0.733, *p* = 0.341) cohorts.

**Conclusion:**

Dixon MRI-based parameters of EOMs, LGs, and IF are useful for differentiating active from inactive TAO. The integration of multiple parameters can further improve staging performance.

**Critical relevance statement:**

In this study, the authors explored the combined value of quantitative parameters of EOMs, IF, and LGs derived from Dixon MRI in staging TAO patients, which can support the establishment of a proper therapeutic plan.

**Key Points:**

- The quantitative parameters of EOMs, LGs, and IF are useful for staging TAO.
- The EOM-SIR_mean_, LG-SIR_mean_, and LG-FF_mean_ values were found to independently correlate with active TAO.
- Joint evaluation of orbital tissue improved the ability to assess TAO activity.

**Graphical Abstract:**

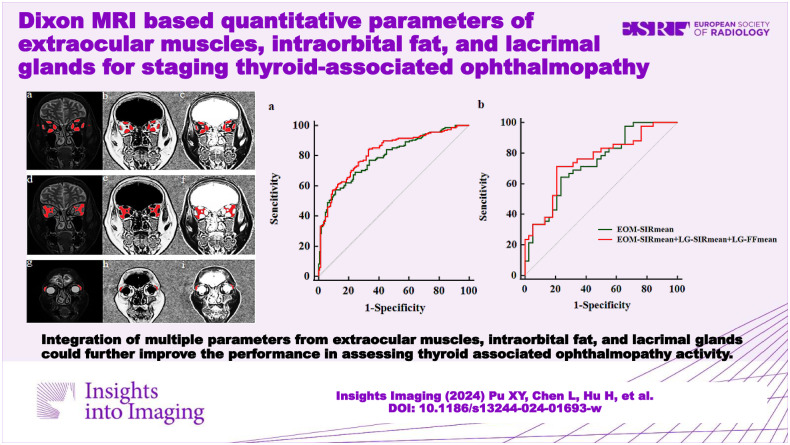

## Introduction

Thyroid-associated ophthalmopathy (TAO) is an autoimmune disorder that affects orbital soft tissues, such as extraocular muscles (EOMs), lacrimal glands (LGs), and intraorbital fat (IF) [1]. Patients with TAO usually experience exophthalmos, eyelid retraction, and diplopia, decreasing quality of life [2, 3]. The natural process of TAO can be divided into two stages: the active stage, which involves inflammatory edema; and the inactive stage, which primarily involves fibrosis and fatty degeneration [4]. The first-line treatment for patients in the active phase is immunosuppressive (e.g., a high dose of intravenous glucocorticoids). By contrast, surgical decompression is usually suggested for patients in the inactive phase [5]. Therefore, it is important to accurately and promptly distinguish between the active and inactive phases for patients with TAO.

The semiquantitative clinical activity score (CAS) is widely used to assess the activity of TAO and predict the response to immunosuppressive treatment [6]. However, the shortcoming of this seven-point scale is its high dependence on the operator’s experience. Moreover, individual muscle involvement cannot be assessed using the CAS alone. Magnetic resonance imaging (MRI), especially fat-suppressed T2-weighted imaging (FS-T2WI), has been widely used to evaluate patients with TAO [7]. Previous studies have indicated that the signal intensity ratios (SIRs) of EOMs, LGs, or IF alone could assist in TAO staging. Higashiyama et al reported that the SIRs of IF and EOMs obtained via FS-T2WI correlated significantly and positively with CAS [8]. Hu et al reported that the SIR of LG on FS-T2WI is a potential imaging biomarker for staging TAO [9]. However, most previous studies have focused on a single structure, and studies combining information on EOMs, LGs, and IF for staging TAO remain scarce.

Conventional FS-T2WI is mainly based on inversion recovery or spectral presaturation, which are prone to imaging artifacts due to magnetic field inhomogeneity at the tissue–air interface between the sinuses and orbit. Severe artifacts can affect the display of EOMs (especially the inferior rectus muscle) and influence staging efficacy [10]. Dixon MRI is a fat-suppressed technique that assesses chemical shift analysis and can directly differentiate fat from water. The superiority of the Dixon technique to conventional inversion recovery or spectral presaturation in terms of overall image quality and FS uniformity has been fully reported [11–13]. However, few studies have been conducted using Dixon MRI to quantitatively assess and integrate data from EOMs, IF, and LGs to stage TAO patients.

Therefore, in this study, we explored the combined value of the quantitative parameters of EOMs, IF, and LGs derived from Dixon MR images for staging patients with TAO.

## Materials and methods

### Patients

**Fig. 1:**
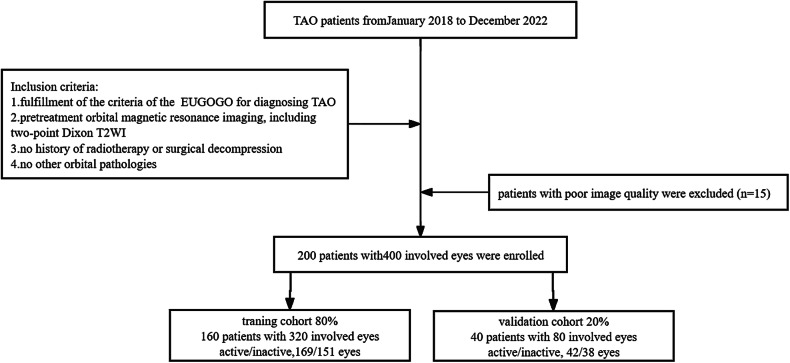
Flowchart of patient enrollment and scheme for analysis. TAO, thyroid-associated ophthalmopathy; EUGOGO, European Group on Graves’ Orbitopathy

This single-center retrospective study was approved by the institutional review board of the First Affiliated Hospital of Nanjing Medical University (Nanjing, China). The requirement for informed consent was waived due to the study’s retrospective nature. All radiological and clinical data were anonymized before analysis. Patients were enrolled from January 2018 to December 2022 according to the following inclusion criteria: (1) fulfilled the criteria of the European Group on Graves’ Orbitopathy (EUGOGO) for diagnosing TAO; (2) included Dixon T2WI in the pretreatment orbital MRI scan; (3) had no history of steroid treatment, radiotherapy, or surgical decompression; and (4) had no other orbital disorders. We identified 215 consecutive patients with TAO in our hospital. Fifteen patients were excluded due to insufficient image quality for further analysis. Finally, a total of 200 patients (121 females; 46.0 ± 13.9 years of age) were included in this study and were divided into training and validation cohorts at a ratio of 8:2 according to the chronological order in which they underwent MR scans. The flowchart of the patient enrollment process is shown in Fig. 1.

### Clinical assessment

Disease activity was assessed for each eye according to the modified seven-point formulation of Mourits’ CAS, which includes the following: (1) spontaneous retrobulbar pain; (2) pain on attempted up or down gaze; (3) redness of the eyelids; (4) redness of the conjunctiva; (5) swelling of the eyelids; (6) inflammation of the caruncle and/or plica; and (7) conjunctival edema [14]. Patients with a CAS of ≥ 3 were enrolled in the active group; otherwise, they were enrolled in the inactive group.

### Image acquisition

All patients were examined using a 3.0-T MRI system (Magnetom Skyra; Siemens Healthcare, Erlangen, Germany) with a 20-channel head coil. The detailed parameters of two-point Dixon T2WI were as follows: repetition time/echo time, 4000/87 ms; field of view, 180 mm; matrix, 179 × 256; section thickness, 3.5 mm; number of excitations, 2; number of sections, 18; and acquisition time (min:s), 2:18.

### Image analysis

All the quantitative parameters of EOMs, LGs, and IF were measured in the unit of each eye. The detailed process was as follows:

1. SIR of EOMs, LGs, and IF to the ipsilateral temporal muscle: three consecutive sections behind the eyeball representing the largest area of the muscle bellies were chosen from coronal water images obtained by Dixon MRI. Polygonal regions of interest (ROIs) were outlined on the superior, inferior, medial, and lateral EOMs using ITK-SNAP software (Fig. 2). Other polygonal ROIs were outlined in two consecutive sections showing the largest slices of the LGs and IF (Fig. 2). The maximum, mean, and minimum signal intensities (SI_max/mean/min_) of the EOMs, IF, and LGs were extracted from PyRadiomics. Moreover, the SI of the ipsilateral temporal muscle was measured using a round ROI of 5–10 mm^2^ using coronal water images obtained by Dixon MRI (Fig. 2). The SIRs of the EOM (EOM-SIR), LG (LG-SIR), and IF (IF-SIR) were calculated using the following formula: SIR_min/mean/max_ = SI_min/mean/max_/SIipsilateral temporal muscle.

**Fig. 2:**
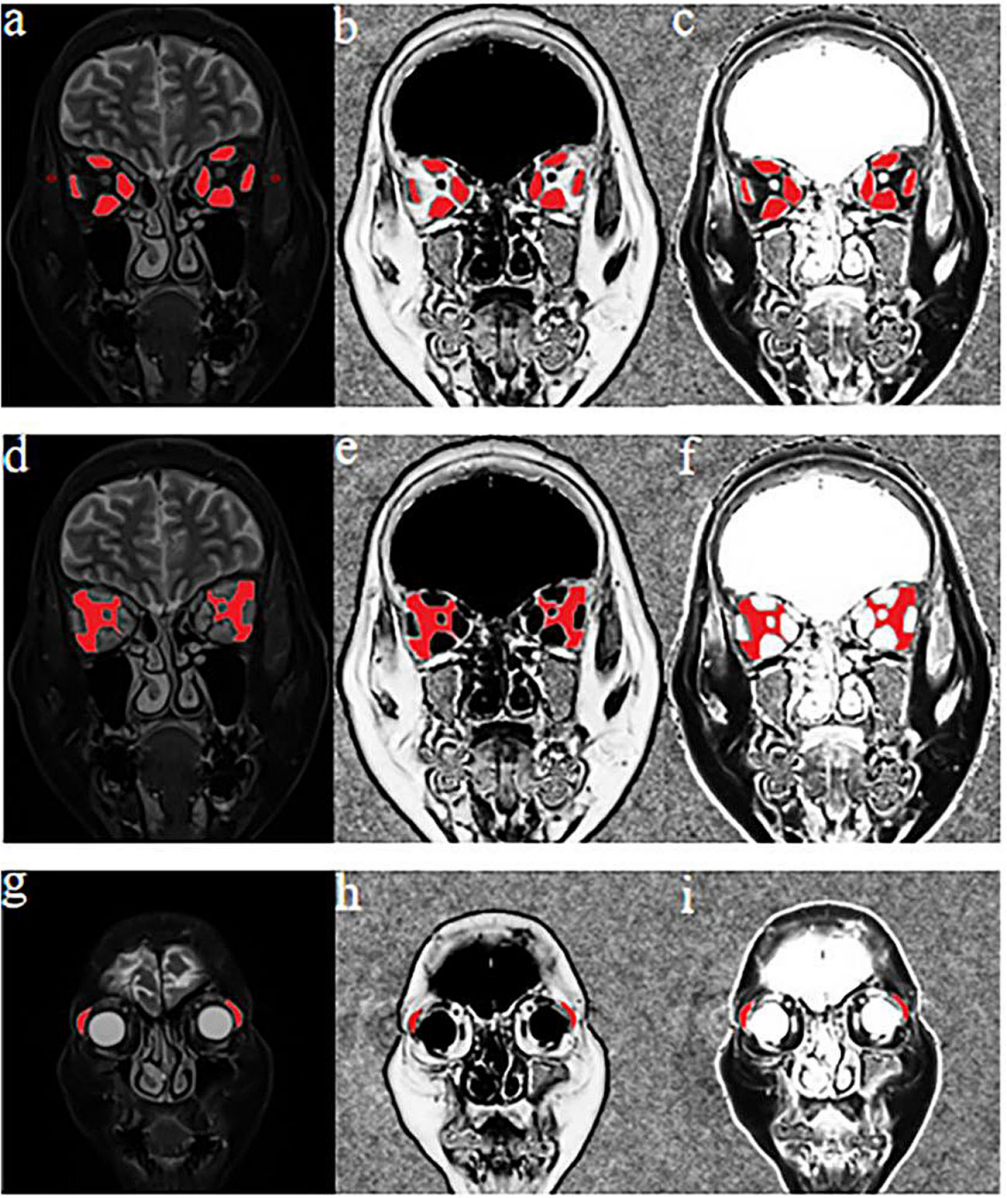
Schematic diagrams showing the methods used to measure the quantitative parameters of EOMs, LGs, and IF using Dixon MRI. T2 Dixon water image (**a**, **d**, **g**), QFFI (**b**, **e**, **h**), and QWFI (**c**, **f**, **i**) of a 54-year-old female with active TAO. **a**–**c** Quantitative measurements of SIR, FF, and WF in the EOM. **a** A circular ROI (red, 5–10 mm^2^) was placed in the ipsilateral temporal muscle. **d**–**f** Quantitative IF measurements of the SIR, FF, and WF. **g**–**i** Quantitative measurements of the SIR, FF, and WF in the LGs. TAO, thyroid-associated ophthalmopathy; QFFI, quantitative fat fraction image; QWFI, quantitative water fraction image; SIR, signal intensity ratio; FF, fat fraction; WF, water fraction; EOMs, extraocular muscle; IF, intraorbital fat; LG, lacrimal gland
2. The water fraction (WF) and fat fraction (FF) of the EOMs, LGs, and IF were calculated as follows: quantitative water fraction images (QWFI) and quantitative fat fraction images (QFFI) were calculated using water and fat images obtained by Dixon MRI in MATLAB software according to the following formula: QWFI = SIwater images/(SIwater images + SIfat images); QFFI = SIfat images/(SIwater images + SIfat images). The abovementioned polygonal ROIs used in the SI measurements were copied into the QWFI and QFFI (Fig. 2). Then, the WF and FF of the EOMs (EOM-WF/FF_min/mean/max_), LGs (LG-WF/FF_min/mean/max_), and IF (IF-WF/FF_min/mean/max_) were obtained by PyRadiomics.

Two radiologists (with 2 and 5 years of experience in neuroradiology) blinded to the study design and clinical information manually and independently selected the ROIs. The measurement results of the two radiologists were used to assess interobserver agreement, and the average value was adopted for further statistical analyses.

### Statistical analyses

The Kolmogorov‒Smirnov test was used to analyze whether the continuous variables were normally distributed. Normally distributed data are reported as the mean ± standard deviation. Otherwise, the data are reported as medians and interquartile ranges. Independent samples *t* tests (normally distributed) or Mann‒Whitney U tests (not normally distributed) were used to compare the continuous variables between the active and inactive groups or the training and validation cohorts. Differences in categorical variables between the two groups were compared using the chi-square test. Significant parameters were included in further binary logistic regression analysis to identify the independent parameters associated with the active stage. The goodness of fit of the logistic regression model was assessed using the Hosmer–Lemeshow test. Logistic regression was used to establish different diagnostic models according to the identified independent parameters. Receiver operating characteristic (ROC) curve analyses and DeLong tests were performed to evaluate and compare the efficiency of different models in staging TAO in both the training and validation cohorts. The interobserver agreement of the quantitative measurements was assessed using the intraclass correlation coefficient (ICC). The ICCs ranged from 0 to 1.00, with values closer to 1.00 indicating better reproducibility. The ICCs were categorized as follows: < 0.40, poor; 0.41–0.60, moderate; 0.61–0.80, good; and ≥ 0.81, excellent [15]. All statistical analyses were conducted using SPSS software (version 25.0; SPSS Inc., Chicago, IL, USA) and MedCalc software (version 18.2.1; MedCalc, Ostend, Belgium). A two-sided *p* value < 0.05 was considered to indicate significance.

## Results

### Clinical characteristics

Among the 200 enrolled patients (400 eyes), 211 eyes had active disease, and the other 189 had inactive disease. The training cohort comprised 160 patients (169 active and 151 inactive eyes), and the validation cohort comprised 40 patients (42 active and 38 inactive eyes). There were no significant differences in demographic or clinical characteristics between the training and validation cohorts (age: 46.1 ± 14.0 vs 45.5 ± 13.4, *p* = 0.755; sex: 60/100 vs 19/21, *p* = 0.102; CAS: 2.5 ± 1.4 vs 2.4 ± 1.1, *p* = 0.583) (Table 1).

**Table 1:** Comparison of patient characteristics between the training and validation cohorts

	Training cohort, (*n* = 160)	Validation cohort, (*n* = 40)	*p*
Age	46.1 ± 14.0	45.5 ± 13.4	0.755
Gender (M/F)	60/100	19/21	0.102
CAS	2.5 ± 1.4	2.4 ± 1.1	0.583
EOM			
SIR			
Max	4.43 ± 1.01	4.63 ± 1.25	0.304
Mean	2.63 ± 0.61	2.73 ± 0.72	0.353
Min	1.11 ± 0.38	1.13 ± 0.49	0.694
FF			
Max	0.57 ± 0.14	0.58 ± 0.16	0.717
Mean	0.08 ± 0.05	0.08 ± 0.06	0.196
Min	0.00 ± 0.00	0.00 ± 0.00	> 0.999
WF			
Max	1.00 ± 0.00	1.00 ± 0.00	> 0.999
Mean	0.92 ± 0.05	0.92 ± 0.06	0.196
Min	0.43 ± 0.14	0.42 ± 0.16	0.717
IF			
SIR			
Max	1.86 ± 0.55	1.85 ± 0.53	0.945
Mean	0.56 ± 0.15	0.56 ± 0.15	0.724
Min	0.00 ± 0.00	0.00 ± 0.00	> 0.999
FF			
Max	1.00 ± 0.00	1.00 ± 0.00	> 0.999
Mean	0.89 ± 0.05	0.90 ± 0.03	0.503
Min	0.41 ± 0.27	0.46 ± 0.24	0.244
WF			
Max	0.59 ± 0.27	0.54 ± 0.24	0.284
Mean	0.11 ± 0.05	0.10 ± 0.03	0.514
Min	0.00 ± 0.00	0.00 ± 0.00	> 0.999
LG			
SIR			
Max	3.62 ± 0.72	3.74 ± 0.72	0.183
Mean	2.57 ± 0.48	2.60 ± 0.53	0.658
Min	1.51 ± 0.52	1.45 ± 0.58	0.634
FF			
Max	0.55 ± 0.18	0.54 ± 0.19	0.825
Mean	0.20 ± 0.12	0.18 ± 0.12	0.406
Min	0.00 ± 0.02	0.00 ± 0.03	0.229
WF			
Max	1.00 ± 0.02	1.00 ± 0.03	0.229
Mean	0.80 ± 0.12	0.82 ± 0.12	0.422
Min	0.45 ± 0.18	0.45 ± 0.20	0.958

The numeric data are reported as the mean ± standard deviation

*n* In parentheses indicates the number of patients

*CAS* clinical activity score, *EOM* extraocular muscle, *IF* intraorbital fat, *LG* lacrimal gland, *SIR* signal intensity ratio, *FF* fat fraction, *WF* water fraction

### Comparisons of Dixon MRI-based quantitative parameters

The interreader reproducibility was good to excellent (ICC, 0.710–0.961) for all Dixon MRI-based quantitative parameters. No significant differences were found between the training and the validation cohorts in any of the Dixon MRI-based quantitative parameters (Table 1). In the training cohort, active TAOs showed significantly greater EOM-SIR_max_ (*p* < 0.001), EOM-SIR_mean_ (*p* < 0.001), EOM-SIR_min_ (*p* < 0.001), IF-SIR_max_ (*p* < 0.001), IF-SIR_mean_ (*p* < 0.001), LG-SIR_max_ (*p* < 0.001), LG-SIR_mean_ (*p* = 0.004), EOM-WF_mean_ (*p* < 0.001), EOM-WF_min_ (*p* < 0.001), IF-WF_max_ (*p* = 0.005), IF-WF_mean_ (*p* < 0.001), and LG-WF_mean_ (*p* = 0.030) values than did inactive TAOs (Table 2). Moreover, active TAOs demonstrated significantly lower EOM-FF_max_ (*p* < 0.001), EOM-FF_mean_ (*p* < 0.001), IF-FF_mean_ (*p* < 0.001), IF-FF_min_ (*p* = 0.011), and LG-FF_mean_ (*p* = 0.030) values than inactive TAOs (Table 2).

**Table 2:** Comparison of Dixon MRI-based quantitative parameters between the active and inactive TAO groups in the training cohort

	Active, (*n* = 169)	Inactive, (*n* = 151)	*p*
EOM			
SIR			
Max	4.86 ± 1.00	3.95 ± 0.78	< 0.001
Mean	2.92 ± 0.61	2.31 ± 0.42	< 0.001
Min	1.22 ± 0.39	0.99 ± 0.34	< 0.001
FF			
Max	0.53 ± 0.14	0.63 ± 0.13	< 0.001
Mean	0.06 ± 0.04	0.11 ± 0.05	< 0.001
Min	0.00 ± 0.00	0.00 ± 0.00	> 0.999
WF			
Max	1.00 ± 0.00	1.00 ± 0.00	> 0.999
Mean	0.94 ± 0.04	0.89 ± 0.05	< 0.001
Min	0.47 ± 0.14	0.37 ± 0.13	< 0.001
IF			
SIR			
Max	2.02 ± 0.58	1.69 ± 0.46	< 0.001
Mean	0.59 ± 0.16	0.52 ± 0.11	< 0.001
Min	0.00 ± 0.00	0.00 ± 0.00	> 0.999
FF			
Max	1.00 ± 0.00	1.00 ± 0.00	> 0.999
Mean	0.89 ± 0.04	0.90 ± 0.06	< 0.001
Min	0.38 ± 0.26	0.45 ± 0.27	0.011
WF			
Max	0.62 ± 0.26	0.55 ± 0.27	0.005
Mean	0.11 ± 0.04	0.10 ± 0.06	< 0.001
Min	0.00 ± 0.00	0.00 ± 0.00	> 0.999
LG			
SIR			
Max	3.77 ± 0.77	3.45 ± 0.62	< 0.001
Mean	2.65 ± 0.49	2.48 ± 0.45	0.004
Min	1.54 ± 0.53	1.47 ± 0.51	0.364
FF			
Max	0.54 ± 0.18	0.56 ± 0.18	0.542
Mean	0.18 ± 0.11	0.22 ± 0.13	0.030
Min	0.01 ± 0.03	0.00 ± 0.00	0.129
WF			
Max	0.99 ± 0.03	1.00 ± 0.00	0.129
Mean	0.82 ± 0.11	0.78 ± 0.13	0.030
Min	0.46 ± 0.18	0.44 ± 0.18	0.542

The numeric data are reported as the mean ± standard deviation

*n* In parentheses indicates the number of patients

*EOM* indicates extraocular muscle, *IF* intraorbital fat, *LG* lacrimal gland, *SIR* signal intensity ratio, *FF* fat fraction, *WF* water fraction

### Logistic regression analysis

**Fig. 3:**
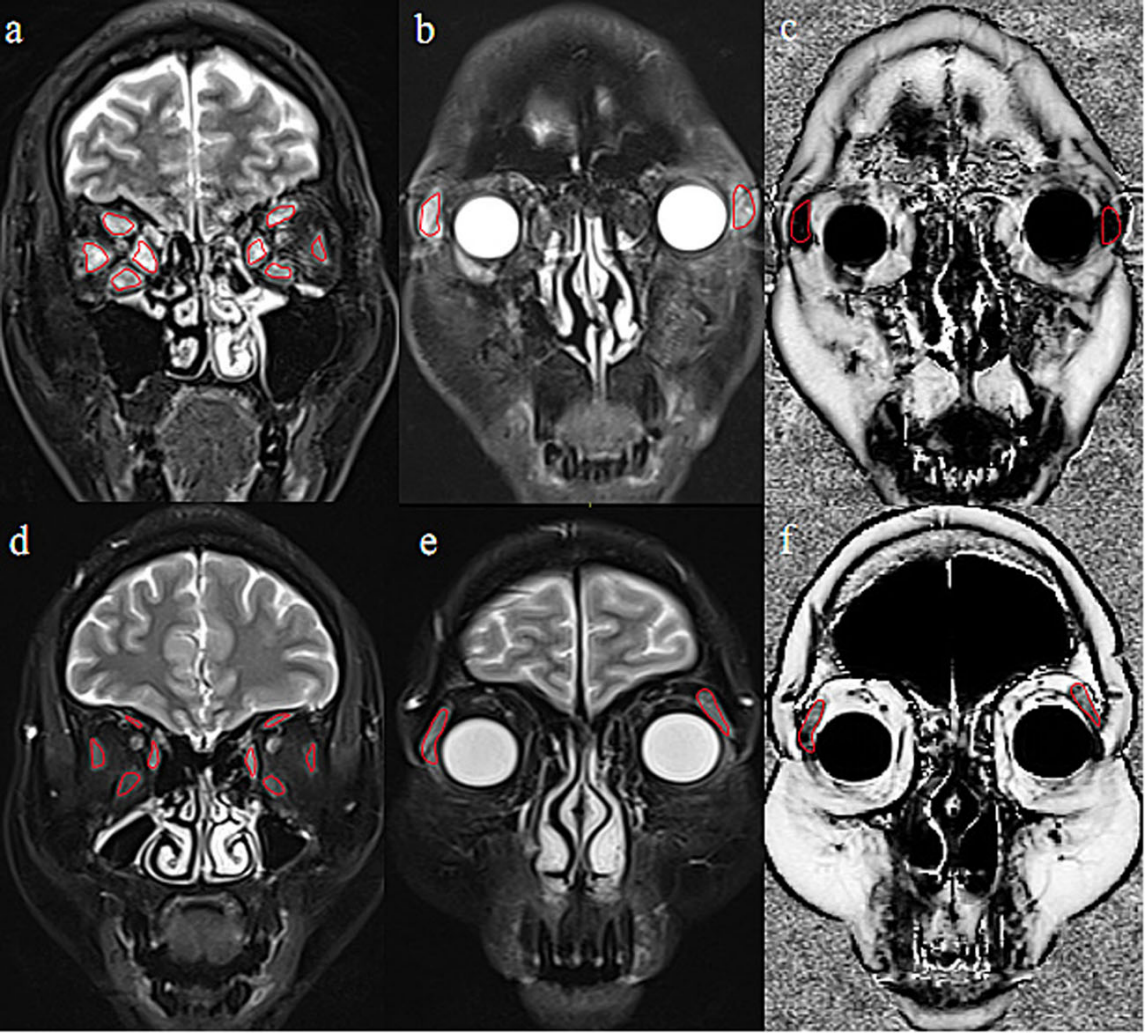
Representative cases of patients with active and inactive TAO. **a**–**c** A 48-year-old man with active TAO and a bilateral CAS of 5. **d**–**f** A 50-year-old woman with inactive TAO and a bilateral CAS of 1. The EOM-SIRmean, LG-SIRmean, and LG-FFmean values were 2.865/3.407, 3.661/3.543, and 0.026/0.019, respectively, in the left/right orbit for patients with active TAO (**a**–**c**) and 2.330/2.082, 2.183/2.002, and 0.392/0.487, respectively, in the left/right orbit for patients with inactive TAO (**d**–**f**)

Binary logistic regression analysis indicated that the EOM-SIR_mean_ (odds ratio [OR] = 18.187, β = 2.901, *p* < 0.001), LG-SIR_mean_ (OR = 0.261, β = −1.341, *p* = 0.001), and LG-FF_mean_ (OR = 0.015, β = −4.230, *p* = 0.003) values were independent predictors of active TAO. Representative patients with active and inactive TAO are presented in Fig. 3.

### ROC curve analysis

**Fig. 4:**
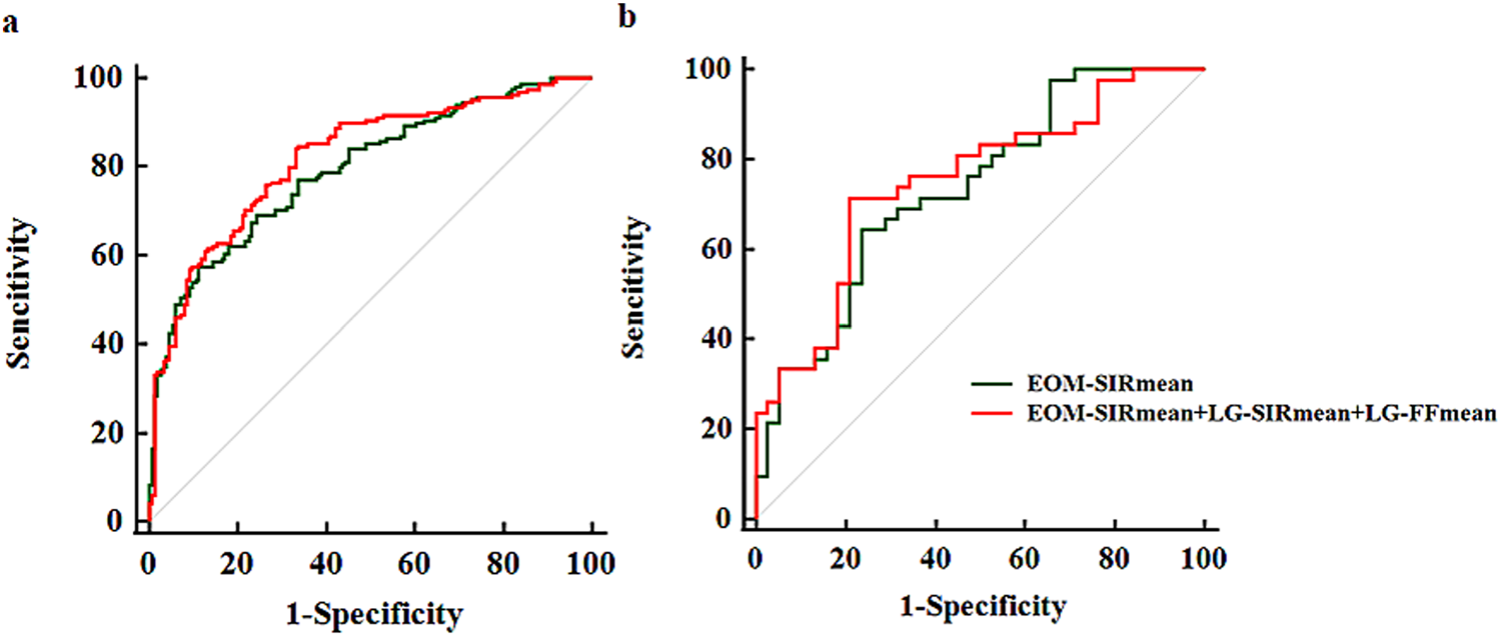
Receiver operating characteristic curves of significant parameters for discriminating active from inactive TAO patients in the training (**a**) and validation (**b**) cohorts

We established two staging models, model 1 (EOM-SIR_mean_ alone) and model 2 (EOM-SIR_mean_ + LG-SIR_mean_ + LG-FF_mean_), according to the logistic regression analysis results. In the training cohort, the optimal performance in staging TAO patients was achieved with model 2, with an area under the curve (AUC) of 0.820, a sensitivity of 84.02%, a specificity of 66.89%, a positive predictive value (PPV) of 74.00%, and a negative predictive value (NPV) of 78.90%. The staging performance of model 2 was significantly better than that of model 1 (AUC, 0.793; sensitivity, 57.40%; specificity, 88.74%; PPV, 85.10%; NPV, 65.00%) (AUC, 0.820 vs 0.793, *p* = 0.016) (Fig. 4).

In the validation cohort, model 2 (AUC, 0.751; sensitivity, 71.43%; specificity, 78.95%; PPV, 78.90%; NPV, 71.40%) also showed relatively better performance than model 1 (AUC, 0.733; sensitivity, 64.29%; specificity, 76.32%; PPV, 75.00%; NPV, 65.90%), although the difference in the AUCs between the two models did not reach significance (AUC, 0.751 vs 0.733, *p* = 0.341) (Fig. 4).

## Discussion

Our study revealed three main findings. First, all the quantitative parameters of EOMs, LGs, and IF based on Dixon MRI showed significant differences between patients with active and inactive TAO. These findings indicate that the EOMs, LGs, and IF demonstrate potential as target organs for staging TAO. Second, the EOM-SIR_mean_, LG-SIR_mean_, and LG-FF_mean_ values were found to be independent predictors of active TAO. Third, compared with a single parameter based on EOMs, a combined model integrating the EOM-SIR_mean_, LG-SIR_mean_, and LG-FF_mean_ values could further improve the performance in staging patients with TAO.

The involvement of EOMs is a known disease process in patients with TAO [16, 17]. In this study, we found that the SIR_min/mean/max_ values of EOMs were significantly greater in active TAOs than those in inactive TAOs, consistent with previous studies [18, 19]. In addition, using the Dixon MRI technique, our study indicated that active TAOs had higher water-related metrics (EOM-WF_mean_ and EOM-WF_min_) and lower fat-related parameters (EOM-FF_max_ and EOM-FF_mean_) than did inactive TAOs. Previous studies have indicated that the active phase of TAO is dominated by inflammatory responses, while the inactive phase of TAO is dominated by fibrosis, fatty infiltration, and collagen deposition [4, 20]. These mechanisms might explain the elevated water-related metrics in active TAOs and the increased fat-related metrics in inactive TAOs.

Increased orbital fat is another major characteristic of TAO [21]. Previously, Potgieser et al reported that a greater volume of orbital fat is associated with a longer duration of TAO [22]; however, they did not analyze the change in the signal intensity of orbital fat. In our study, the SIR_mean/max_, FF_mean/min_, and WF_mean/max_ values of orbital fat differed significantly between active and inactive TAOs. Previous studies have revealed that orbital fat is histologically characterized by lymphocytic infiltration and edema due to the accumulation of hydrophilic interstitial glycosaminoglycans [23]. We suspect that this accumulation is potentially the mechanism underlying the increased SIR and WF values in patients with active TAO.

As LGs are another potential target organ, changes in LGs in patients with TAO have attracted increasing attention [24]. Gagliardo et al reported that patients with right and left active TAO demonstrated significantly greater herniation of the LGs on MRI than in those with inactive TAO [25]. Using the T2 mapping technique, Jiang et al reported that the T2 mapping values of LGs differed significantly between active and inactive TAO. Together with clinical indicators, the T2 mapping technique could effectively stage patients with TAO [26]. In addition, using the diffusion tensor imaging technique, Chen et al reported that the LGs of active TAO showed significantly lower fractional anisotropy and a higher apparent diffusion coefficient than those of inactive TAO [27]. In our study, similar to the change in EOMs, we found that the LGs of active TAOs had higher SIR_mean/max_ and WF_mean_ values and lower FF_mean_ values. The abovementioned pathological changes in EOMs and IFs could help explain these findings. In addition, two LG-based parameters (LG-SIR_mean_ and LG-FF_mean_) were found to be independently associated with TAO activity. Our results confirmed that the LGs are involved in the TAO process and deserve further study.

According to the binary logistic regression analysis, the EOM-SIR_mean_, LG-SIR_mean_, and LG-FF_mean_ values were found to be independent predictors of active TAO. No IF-related metric was found to be an independent variable, possibly due to our study population’s specific sample size and constitution. Furthermore, we constructed a predictive model by integrating the LG-SIR_mean_ and LG-FF_mean_ on the basis of the EOM-SIR_mean_ for staging patients with TAO. The ROC analysis results indicated that the combined model outperformed the EOM-SIR_mean_ alone in both the training and validation cohorts. These results indicated that information on EOMs and other target organs (e.g., LGs and IF) should be integrated and analyzed for staging TAO. Further multicenter studies with larger sample sizes are needed to confirm our results and establish a more robust model for staging patients with TAO in clinical practice.

Our study has several limitations. First, this was a retrospective study from a single center. Further studies with larger study populations and external validation are needed to confirm the findings presented here. Second, the exact pathological state of orbital tissues remains unclear due to the difficulty in obtaining histological samples from patients with TAO, especially those with active disease. Future studies to determine the correlations between imaging metrics and histological changes are needed [28]. Third, this study focused only on the usefulness of the Dixon MRI sequence in staging TAO, and other functional MR sequences (e.g., diffusion or mapping sequences) were not simultaneously scanned. Further studies using machine learning methods to integrate more information from more functional sequences could further improve staging performance.

In conclusion, our study showed that the quantitative parameters of EOMs, LGs, and IF derived from Dixon MR images are useful for differentiating active from inactive TAOs. Integrating multiple parameters from EOMs, LGs, and IF could further improve TAO patient staging.

## Data Availability

The data used to support the findings of this study are available from the corresponding author upon request.

## Abbreviations

AUC: Area under the curve
CAS: Clinical activity score
EOM: Extraocular muscle
FF: Fat fraction
ICC: Intraclass correlation coefficient
IF: Intraorbital fat
LG: Lacrimal gland
NPV: Negative predictive value
PPV: Positive predictive value
QFFI: Quantitative fat fraction image
QWFI: Quantitative water fraction image
ROC: Receiver operating characteristic
ROI: Region of interest
SIR: Signal intensity ratio
TAO: Thyroid-associated ophthalmopathy
WF: Water fraction

## References

[1] Bartalena L, Tanda ML (2022). Current concepts regarding Graves’ orbitopathy. J Intern Med.

[2] Yu CY, Ford RL, Wester ST, Shriver EM (2022). Update on thyroid eye disease: regional variations in prevalence, diagnosis, and management. Indian J Ophthalmol.

[3] Bahn RS (2010). Graves’ ophthalmopathy. N Engl J Med.

[4] Bartalena L, Piantanida E, Gallo D, Lai A, Tanda ML (2020). Epidemiology, natural history, risk factors, and prevention of Graves’ orbitopathy. Front Endocrinol (Lausanne).

[5] Bartalena L, Kahaly GJ, Baldeschi L (2021). The 2021 European group on Graves’ Orbitopathy (EUGOGO) clinical practice guidelines for the medical management of Graves’ orbitopathy. Eur J Endocrinol.

[6] Mourits MP, Prummel MF, Wiersinga WM, Koornneef L (1997). Clinical activity score as a guide in the management of patients with Graves’ ophthalmopathy. Clin Endocrinol (Oxf).

[7] Mayer EJ, Fox DL, Herdman G (2005). Signal intensity, clinical activity and cross-sectional areas on MRI scans in thyroid eye disease. Eur J Radiol.

[8] Higashiyama T, Iwasa M, Ohji M (2017). Quantitative analysis of inflammation in orbital fat of thyroid-associated ophthalmopathy using MRI signal intensity. Sci Rep.

[9] Hu H, Xu XQ, Wu FY (2016). Diagnosis and stage of Graves’ ophthalmopathy: efficacy of quantitative measurements of the lacrimal gland based on 3-T magnetic resonance imaging. Exp Ther Med.

[10] Delfaut EM, Beltran J, Johnson G, Rousseau J, Marchandise X, Cotten A (1999). Fat suppression in MR imaging: techniques and pitfalls. Radiographics.

[11] Chen L, Hu H, Chen HH (2021). Usefulness of two-point Dixon T2-weighted imaging in thyroid-associated ophthalmopathy: comparison with conventional fat saturation imaging in fat suppression quality and staging performance. Br J Radiol.

[12] Sun J, Xing Z, Chen J (2018). Fat status detection and histotypes differentiation in solid renal masses using Dixon technique. Clin Imaging..

[13] Kox LS, Kraan RBJ, Mazzoli V (2020). It’s a thin line: development and validation of Dixon MRI-based semi-quantitative assessment of stress-related bone marrow edema in the wrists of young gymnasts and non-gymnasts. Eur Radiol.

[14] Bartalena L, Baldeschi L, Boboridis K (2016). The 2016 European Thyroid Association/European Group on Graves’ Orbitopathy guidelines for the management of Graves’ orbitopathy. Eur Thyroid J.

[15] Chen W, Hu H, Chen HH (2020). Utility of T2 mapping in the staging of thyroid-associated ophthalmopathy: efficiency of region of interest selection methods. Acta Radiol.

[16] Regensburg NI, Wiersinga WM, Berendschot TT, Potgieser P, Mourits MP (2011). Do subtypes of graves’ orbitopathy exist?. Ophthalmology.

[17] Song C, Luo Y, Yu G, Chen H, Shen J (2022). Current insights of applying MRI in Graves’ ophthalmopathy. Front Endocrinol (Lausanne).

[18] Li Z, Luo Y, Feng X (2023). Application of multiparameter quantitative magnetic resonance imaging in the evaluation of Graves’ ophthalmopathy. J Magn Reson Imaging..

[19] Liu X, Su Y, Jiang M (2021). Application of magnetic resonance imaging in the evaluation of disease activity in Graves’ ophthalmopathy. Endocr Pract.

[20] Bartalena L, Pinchera A, Marcocci C (2000). Management of Graves’ ophthalmopathy: reality and perspectives. Endocr Rev.

[21] Kuriyan AE, Woeller CF, O’Loughlin CW, Phipps RP, Feldon SE (2013). Orbital fibroblasts from thyroid eye disease patients differ in proliferative and adipogenic responses depending on disease subtype. Invest Ophthalmol Vis Sci.

[22] Potgieser PW, Wiersinga WM, Regensburg NI, Mourits MP (2015). Some studies on the natural history of Graves’ orbitopathy: increase in orbital fat is a rather late phenomenon. Eur J Endocrinol.

[23] Kaichi Y, Tanitame K, Terada H (2019). Thyroid-associated orbitopathy: quantitative evaluation of the orbital fat volume and edema using IDEAL-FSE. Eur J Radiol Open.

[24] Allam IY, Lazreg S, Shafik Shaheen M, Doheim MF, Mohammed MA (2021). Ocular surface changes in patients with thyroid eye disease: an observational clinical study. Clin Ophthalmol.

[25] Gagliardo C, Radellini S, Morreale Bubella R (2020). Lacrimal gland herniation in Graves ophthalmopathy: a simple and useful MRI biomarker of disease activity. Eur Radiol.

[26] Jiang M, Song X, Zhang H (2022). The combination of T2-mapping value of lacrimal gland and clinical indicators can improve the stage prediction of Graves’ ophthalmopathy compared to clinical activity scores. Endocrine.

[27] Chen L, Hu H, Chen W (2021). Usefulness of readout-segmented EPI-based diffusion tensor imaging of lacrimal gland for detection and disease staging in thyroid-associated ophthalmopathy. BMC Ophthalmol.

[28] Hu H, Chen L, Zhou J (2022). Multiparametric magnetic resonance imaging for differentiating active from inactive thyroid-associated ophthalmopathy: added value from magnetization transfer imaging. Eur J Radiol.

